# Supplementary material for: Designing multimedia patient education materials for adolescent idiopathic scoliosis: A protocol for a feasibility randomized controlled trial of patient education videos
Source: PLoS One. 2024 May 23;19(5):e0297394. doi: 10.1371/journal.pone.0297394 (PMC11115215; doi:10.1371/journal.pone.0297394)
Supplement: S1 File — (PDF) [file pone.0297394.s002.pdf]

HREC SUPPORTING DOCUMENT CHECKLIST & TEMPLATE
for submission via InfoHub

This template is a checklist and intended to aid your submission to the HREC for a full ethical review by providing you with a reminder of all the documents you *might* submit in one file. All supporting documents, applicable to your study, should be inserted into this document where indicated. **Please note that your submission cannot be reviewed without the relevant Information Sheet(s) and Consent/Assent Form(s). Section 8 is for responses to decision points after the review – not for new submissions.**

Please indicate** (by double clicking tick box and selecting 'checked')* ***the documents you have provided for review only

SUPPORT DOCUMENTS		
**1. Information Sheets**		
a)	Information Sheet for Participants	Yes ☒
b)	Information Sheet for Parents/Guardians/Other	Yes ☒
c)	Information Sheet for Children *(age appropriate text)*	Yes ☒
**2. Consent & Assent Forms**		
a)	Consent Form for Participants	Yes ☒
b)	Consent Form for Parents/Guardians/Other	Yes ☒
c)	Assent Form for Children *(under 18)*	Yes ☒
**3. Interviews, Questionnaires, Surveys & Recruitment**		
a)	Interview Schedule for Interviews/Focus Groups	Yes ☐
b)	Questionnaires/Surveys	Yes ☒
c)	Advertisement/Poster/flyers for recruitment of participants	Yes ☒
**4. Permissions & Endorsements**		
a)	Letter of endorsement from your supervisor *(if applicable)* this should be a pdf which can either be included in this document or sent separately by email	Yes ☒
b)	Copy of Garda Vetting Certificate *(if applicable)* this should be a pdf with which can either be included in this document or uploaded to Infohub separately or sent by email.	Yes ☒
c)	Letter(s) of permission/approval from external organization(s) granting access to their business/school/charity/database (secondary data) etc.,	Yes ☒
**5. Additional Relevant Supporting Documents** *(see page 12 for examples)*		Yes ☒
**6. Human Research Risk Assessment** for all face -to-face data health data collection you will be required to complete a self-assessment form and this **may** be requested as part of your review. Please see https://www.ucd.ie/sirc/coronavirus/returntocampusworking/		***Not to be included in this template***
**7. Insurance for Human Research** applicants should use the mandatory self-assessment checklist https://www.ucd.ie/sirc/insurance Every applicant must ensure that the appropriate insurance cover is in place for their research. The HREC is not responsible for overseeing insurance requirements.		***Not to be included in this template***
**8. Post Review only – not required for new submissions** *(see page 13 for clarification)*		Yes ☐

***Insert all Information Sheets here** (participants, parents/guardians/ teachers/other) and ensure that they following the correct format – see **Question 11** in the HREC Application Form (HR1) – please confirm that you will print this document on your School Headed Paper*

PARTICIPANT INFORMATION SHEET (under 18)

Thank you for your interest in this study. This is a study about using videos to teach people about scoliosis. My name is Garett Van Oirschot, and I am a student in the UCD School of Public Health, Physiotherapy & Sports Science.

What is this research about?
This research is about teaching patients about Adolescent Idiopathic Scoliosis. We want to see if different ways of teaching can help patients learn better or help with their health.

Why are we doing this research?
We want to check how much patients know about scoliosis, or what they have learned from healthcare workers already.
We also might show some people different videos about scoliosis, based on ones you find online now and compare to ones we make at UCD.

Why have we asked you?
You were asked because

- You are 10-18 years old
- You have Adolescent Idiopathic Scoliosis

What happens if I volunteer?
If you volunteer for the study, we will track how you learn and understand information and you will have to answer some questions online. You will need to remember information from your doctor or physio before doing a weekly quiz, then doing a final quiz after the six weeks. We want to ask you what you know about scoliosis.
Two of the treatment groups will be asked to watch a video each week that is less than 3 minutes, and answer 5 questions every week on a computer or phone or tablet for six weeks, then answer the questions again when the study finishes after eight weeks, and then again six months later.

After the study is finished, the data will be used for a PhD thesis and may be shared with other researchers. This will help us to understand how to teach people with Adolescent Idiopathic Scoliosis.

You can also change your mind and quit the study at any time, and you do not need parent/guardian permission to quit they study.

How will your data and privacy be protected?

Your identity will be kept secret by one of the researchers and your answers will not have your name on them. Once the study over, we will destroy the secret codesheet and the data cannot be traced back to you.

What are the benefits and risks?

If you participate, you will be helping researchers learn about the best way to teach people just like you.

You will learn more about your condition, but there is a risk that it may make you upset, so a Physiotherapist will be ready to help you if needed. We can also get help from other health workers who deal with young people who feel upset, or with health workers who can answer questions scoliosis.

You can talk to a Physiotherapist on the research team or any other healthcare provider if you have any questions or concerns.

How will I find out what happens in the study?

After the study is finished, we will share the results with other researchers as well as online

If you want to know more, you can ask the research team. They will be happy to help you understand the results better.

Who can I speak to about volunteering? Or to get more information?

If you decide to volunteer for this study, or if you want more information, please feel free to contact:

Garett Van Oirschot MMSkSportPhty, BScPhysio
PhD student in UCD School of Public Health, Physiotherapy & Sports Science
CORU Registered Physiotherapist PT030804
E: [redacted]
M: [redacted]

PARTICIPANT INFORMATION SHEET (Age 18)

Thank you for your interest in this study of multimedia education for patients with Adolescent Idiopathic Scoliosis. I (Garett Van Oirschot) am undertaking this research as part of a PhD in the UCD School of Public Health, Physiotherapy & Sports Science.

What is this research about?

Patients are often provided with education about their condition and while much research goes into deciding the content of the education, less research has been done on format and design. I am conducting this research with the goal of testing different formats of education for patients with Adolescent Idiopathic Scoliosis.

Why are we doing this research?

To determine how different types of design affect patient learning, engagement, and other scoliosis-related outcomes. We want to compare the education you receive from healthcare providers with multimedia information found online, and also with multimedia information that we have designed here at UCD.

Why hve you been invited?

You have been invited because you are

- Aged 10-18
- Diagnosed with Adolescent Idiopathic Scoliosis, confirmed via X-ray

What will happen if I volunteer?

We would like to track your learning about scoliosis. We would like you to answer some questionnaires every week for six weeks, as you receive information from health professionals, information you might find online, or information that we might provide, depending on which group you are randomised into.
You may be asked watch a maximum 3-minute informational video on a computer, phone or tablet each week. After this we will ask the same questions again, and then again after six months, and then the study will be complete.

How will my data be used and privacy protected?

This research will be carried out in compliance with GDPR regulations, meaning your personal data will not be shared without your consent.
You will have contact with a Physiotherapist on the research team who will keep all your data confidential.
Your answers on any questionnaires will be kept confidential and made anonymous to the researcher who analyses the survey data.

All data will be de-identified and coded with a unique ID number that only the main researcher has access to, so that it cannot be traced back to you. After the study is completed then the master sheet linking your name and ID number will be destroyed, making all the data permanently anonymous and it will no longer be possible to withdraw from the study because at this stage the data cannot be linked to you.
Results will be stored in UCD and used as part of a PhD thesis and submitted as an article for a science journal, and this anonymous data could potentially be made available to other researchers in the future.

What are the benefits and risks if I participate?

Your participation in the study will increase the understanding of how to provide information to people diagnosed with Adolescent Idiopathic Scoliosis. You will also be provided with education about Adolescent Idiopathic Scoliosis.
There is a risk you could become upset by learning information about Adolescent Idiopathic Scoliosis, or have questions that are not answered by the information provided. Therefor each time that you are presented with information, a Physiotherapist on the research team can be contacted to answer questions and address any concerns. We can also refer you to a healthcare professional in the field of mental health, orthopaedics or a support network like spunout.ie or jigsaw.ie or, if you wish, we can also pass these questions or concerns along to any other healthcare providers of your choice.

Can I change my mind at any stage and withdraw from the study?
Absolutely.

Confidentiality and consent
Your identity in the published research will remain confidential. Your name and personal details will not be disclosed. As noted above, following the study your identifiable information will be permanently removed so you will no longer be able to withdraw.
This study is completely voluntary. You can change your mind to participate at any stage during the study and withdraw without prejudice.

How will we find out what happens in the study?
After the study, the results will be written up for publication in a scientific journal. The results will also be made available and shared in plain English over social media so you can find out what happens. If you wish to know more, please let us know and the researcher team can inform you when the project is finished.

Conflicts of interest
A Physiotherapist on the research team treats patients with Adolescent Idiopathic Scoliosis in private practice, treating patients with scoliosis.

Has this study been reviewed by an Ethics Committee?
Ethical approval has been granted by the UCD Human Research Ethics Committee (HERC) for this study as file number ______.

Who can we speak to about volunteering? Or to get more information?
If you have decided to volunteer for this study, or if you would like more information please feel free to contact:

Garett Van Oirschot MMSkSportPhty, BScPhysio
PhD student in UCD School of Public Health, Physiotherapy & Sports Science
CORU Registered Physiotherapist PT030804
E: [redacted]
M: [redacted]

PARENT/GUARDIAN INFORMATION SHEET

Thank you for your interest in this study of multimedia education for patients with Adolescent Idiopathic Scoliosis. I (Garett Van Oirschot) am undertaking this research as part of a PhD in the UCD School of Public Health, Physiotherapy & Sports Science.

What is this research about?

Patients are often provided with education about their condition and while much research goes into deciding the content of the education, less research has been done on format and design. I am conducting this research with the goal of testing different formats of education for patients with Adolescent Idiopathic Scoliosis.

Why are we doing this research?

To determine how different types of design affect patient learning, engagement, and other scoliosis-related outcomes. We want to compare the education you receive from healthcare providers with multimedia information found online, and also with multimedia information that we have designed here at UCD.

Why has my child been invited?

Your child has been invited because you are

- Aged 10-18
- Diagnosed with Adolescent Idiopathic Scoliosis, confirmed via X-ray

What will happen if my child volunteers?

We would like to track your child's learning about scoliosis. We would like your child to answer some questionnaires every week for six weeks, as they receive information from health professionals, information you might find online, or information that we might provide, depending on which group they are randomised into. **Your child may be asked watch a maximum 3-minute informational video on a computer, phone or tablet each week. After this we will ask the same questions again at eight weeks, and then again at six months, and then the study will be complete.**

How will my child's data be used and privacy protected?

This research will be carried out in compliance with GDPR regulations, meaning your personal data will not be shared without your consent.

You and your child will be in contact with a Physiotherapist on the research team who will keep all your data confidential.

Your child's answers on any questionnaires will be kept confidential and made anonymous to the researcher who collects the survey data.

All data will be de-identified and coded with a unique ID number that only the main researcher has access to, so that it cannot be traced back to your child. After the study is completed then the master sheet linking your child's name and number will be destroyed, making all the data permanently anonymous and it will no longer be possible to withdraw from the study because at this stage the data is no longer linked to your child. It will be stored in UCD and used as part of a PhD thesis and submitted as an article for a science journal, and this anonymous data could potentially be made available to other researchers in the future.

What are the benefits and risks if my child participates?

Your child's participation in the study will increase the understanding of how to provide information to people diagnosed with Adolescent Idiopathic Scoliosis. Your child will also be provided with education about Adolescent Idiopathic Scoliosis.
There is a risk your child could become upset by learning information about Adolescent Idiopathic Scoliosis, or have questions that are not answered by the information provided. Therefor each time that your child is presented with information, a Physiotherapist on the research team can be contacted to answer questions and address any concerns. We can also refer you and your child to a healthcare professional in the field of mental health, orthopaedics or a support network like spunout.ie or jigsaw.ie or, if you wish, we can also pass these questions or concerns along to any other healthcare providers of your choice.

Can my child change your mind at any stage and withdraw from the study?
Absolutely.

Confidentiality and consent
Your child's identity in the published research will remain confidential. Your name and personal details will not be disclosed. As noted above, following the study your child's identifiable information will be removed so your child will no longer be able to withdraw.
This study is completely voluntary. You or your child can change your mind to participate at any stage during the study and withdraw without prejudice.

How will we find out what happens in the study?
After the study, the results will be written up for publication in a scientific journal. The results will also be made available and shared in plain English over social media so you can find out what happens. If you wish to know more, please let us know and the researcher team can inform you when the project is finished.

Conflicts of interest
A Physiotherapist on the research team treats patients with Adolescent Idiopathic Scoliosis in private practice, treating patients with scoliosis.

Has this study been reviewed by an Ethics Committee?
Ethical approval has been granted by the UCD Human Research Ethics Committee (HERC) for this study as file number ______.

Who can we speak to about volunteering? Or to get more information?
If your child has decided to volunteer for this study, or if you would like more information please feel free to contact:

Garett Van Oirschot MMSkSportPhty, BScPhysio
PhD student in UCD School of Public Health, Physiotherapy & Sports Science
CORU Registered Physiotherapist PT030804
E: [redacted]
M: [redacted]

Insert all consent forms here *(participants, parents/guardians/ teachers/other) – the format can vary as researchers may want to itemize everything that they need a participant to consent to that is involved in the current study, and may anticipate further research such as future publications, archiving or re-using the de-identified data at a later stage. Please also confirm here that you will print this document on your School's Headed Paper.*

PARENT/GUARDIAN CONSENT FORM:

PLEASE TICK YOUR RESPONSE IN THE APPROPRIATE BOX

I have read and understood the Participant Information Sheet
YES ☐ NO ☐
I have had the opportunity to ask questions and discuss the study
YES ☐ NO ☐
I have received satisfactory answers to all my questions
YES ☐ NO ☐
I have received enough information about this study
YES ☐ NO ☐
I consent to my child's data being stored digitally and archived at the end of the study
YES ☐ NO ☐
I consent to my child's data being shared with UCD research partners in future
YES ☐ NO ☐
I understand that my child is free to withdraw from the study at any time without giving a reason
YES ☐ NO ☐
I agree for my child to take part in the study
YES ☐ NO ☐

Parent/Guardian Signature: ______________________________ Date: _________

Parent/Guardian Name in print: ____________________________

Investigator's Signature: _____________________________ Date: _________

Investigator's Name in print: __________________________

If you would like more information, please feel free to contact:
Garett Van Oirschot MMSkSportPhty, BScPhysio
PhD student in UCD School of Public Health, Physiotherapy & Sports Science
CORU Registered Physiotherapist PT030804
E: [redacted]
M: [redacted]

PARTICIPANT CONSENT FORM (age 18)

Did you read and understand the information about this study?
YES ☐ NO ☐
Did you have a chance to ask questions and talk about this study?
YES ☐ NO ☐
Did you get answers to all your questions?
YES ☐ NO ☐
Did you get enough information about this study?
YES ☐ NO ☐
Can we save your study information on a computer and keep it after this study is over?
YES ☐ NO ☐
Can we share your study information with other groups who study at the University College Dublin?
YES ☐ NO
Do you understand that you can exit the study at any time and you don't have to tell us why?
YES ☐ NO ☐
Do you agree to be in the study?
YES ☐ NO ☐

Signature: ______________________________ Date: __________

Name in print: ____________________________

Investigator's Signature: _____________________________ Date: __________

Investigator's Name in print: __________________________

If you have any more questions, please contact:

Garett Van Oirschot MMSkSportPhty, BScPhysio
PhD student in UCD School of Public Health, Physiotherapy & Sports Science
CORU Registered Physiotherapist PT030804
E: [redacted]
M[redacted]

Insert ***Children's Assent Form****, if applicable, here and ensure that it is written in age-appropriate language*

PARTICIPANT ASSENT FORM (under age 18)

Did you read and understand the information about this study?
YES ☐ NO ☐
Did you have a chance to ask questions and talk about this study?
YES ☐ NO ☐
Did you get answers to all your questions?
YES ☐ NO ☐
Did you get enough information about this study?
YES ☐ NO ☐
Can we save your study information on a computer and keep it after this study is over?
YES ☐ NO ☐
Can we share your study information with other groups who study at the University College Dublin?
YES ☐ NO ☐
Do you understand that you can exit the study at any time and you don't have to tell us why?
YES ☐ NO ☐
Do you agree to be in the study? You can say no even if your parent/guardian said yes.
YES ☐ NO ☐

Signature: ______________________________ Date: _________

Name in print: ____________________________

Investigator's Signature: _____________________________ Date: _________

Investigator's Name in print: __________________________

If you have any more questions, please contact:

Garett Van Oirschot MMSkSportPhty, BScPhysio
PhD student in UCD School of Public Health, Physiotherapy & Sports Science
CORU Registered Physiotherapist PT030804
E: [redacted]
M: [redacted]

Insert ***Recruitment Advertisement/Poster or flyers here*** *– if the document is not in Word please insert the text only*

Volunteers needed!

Are you aged <u>10-18 years</u>?
Have you been diagnosed with <u>scoliosis</u>?

We are researching educational materials that are used to teach about scoliosis…

Are you be able to:

- attend an introductory meeting online or in person?
- spend at least 3 minutes per week watching a video based on information from the Scoliosis Research Society?
- do a survey about what you have watched or read?

If yes, than we would love to hear from you!

[redacted] [redacted] [redacted] [redacted] [redacted] [redacted] [redacted]

Social Media graphic:

[redacted]

UCD School of Public Health, Physiotherapy and Sports Science

Scoil na Sláinte Poiblí, na Fisiteiripe agus Eolaíocht an Daonra UCD

Scoliosis Research

Are you aged <u>10-18</u>?
Do you have <u>scoliosis</u>?

We are researching educational materials used to teach about scoliosis...

Are you be able to:

- Attend an introductory meeting online or in person?
- Watch a weekly 3-minute video based on information from the Scoliosis Research Society, plus a weekly 5-question quiz?
- Do a 20-minute survey about yourself before the study, after the study, and again 6 months after the study?

If yes, please click below!

Insert ***Questionnaires/Surveys/scales*** *and any associated evaluation document here and confirm that you have permission to use copyrighted questionnaires.*

Permission to use the EQ-5D-Y:

Dear Mr. Garett Van Oirschot ,

Thank you for your registration.

The study / project titled "Multimedia Education in Adolescent Idiopathic Scoliosis: A randomised controlled trial".you registered fulfills the conditions for you to use the requested version(s) free of charge.

Below you find our Terms of Use Non-Commercial. We will provide you with the requested versions free of charge once we have received your agreement with our Terms of Use. You can indicate your agreement by pressing the green " Agree" button below. If you do not agree, please press " Disagree" .

If you have any questions please contact us by sending an email to [redacted]

Thank you in advance.

Kind regards,

SRS-22r Patient Questionnaire

Patient Name: ____________ ________ ______________ Date of Birth:____ _____ ____
First MI Last Mo Day Yr

Today's Date: ____ ______ ________ Age: _____+_______
Mo Day Yr Yrs Mo

Medical Record #: ___________________

INSTRUCTIONS: We are carefully evaluating the condition of your back and it is **IMPORTANT THAT YOU ANSWER EACH OF THESE QUESTIONS YOURSELF.** Please **CIRCLE THE ONE BEST ANSWER TO EACH QUESTION.**

1. Which one of the following best describes the amount of pain you have experienced during the past 6 months?

   None
   Mild
   Moderate
   Moderate to severe
   Severe

2. Which one of the following best describes the amount of pain you have experienced over the last month?

   None
   Mild
   Moderate
   Moderate to severe
   Severe

3. During the past 6 months have you been a very nervous person?

   None of the time
   A little of the time
   Some of the time
   Most of the time
   All of the time

(CONTINUED ON NEXT PAGE)

4. If you had to spend the rest of your life with your back shape as it is right now, how would you feel about it?

Very happy
Somewhat happy
Neither happy nor unhappy
Somewhat unhappy
Very unhappy

5. What is your current level of activity?

Bedridden
Primarily no activity
Light labor and light sports
Moderate labor and moderate sports
Full activities without restriction

6. How do you look in clothes?

Very good
Good
Fair
Bad
Very bad

7. In the past 6 months have you felt so down in the dumps that nothing could cheer you up?

Very often
Often
Sometimes
Rarely
Never

8. Do you experience back pain when at rest?

Very often
Often
Sometimes
Rarely
Never

9. What is your current level of work/school activity?

100% normal
75% normal
50% normal
25% normal
0% normal

(CONTINUED ON NEXT PAGE)

10. Which of the following best describes the appearance of your trunk; defined as the human body except for the head and extremities?

    Very good
    Good
    Fair
    Poor
    Very Poor

11. Which one of the following best describes your pain medication use for back pain?

    None
    Non-narcotics weekly or less (e.g., aspirin, Tylenol, Ibuprofen)
    Non-narcotics daily
    Narcotics weekly or less (e.g. Tylenol III, Lorcet, Percocet)
    Narcotics daily

12. Does your back limit your ability to do things around the house?

    Never
    Rarely
    Sometimes
    Often
    Very Often

13. Have you felt calm and peaceful during the past 6 months?

    All of the time
    Most of the time
    Some of the time
    A little of the time
    None of the time

14. Do you feel that your back condition affects your personal relationships?

    None
    Slightly
    Mildly
    Moderately
    Severely

(CONTINUED ON NEXT PAGE)

15. Are you and/or your family experiencing financial difficulties because of your back?

    Severely
    Moderately
    Mildly
    Slightly
    None

16. In the past 6 months have you felt down hearted and blue?

    Never
    Rarely
    Sometimes
    Often
    Very often

17. In the last 3 months have you taken any days off of work, including household work, or schoo because of back pain?

    0 days
    1 day
    2 days
    3 days
    4 or more days

18. Does your back condition limit your going out with friends/family?

    Never
    Rarely
    Sometimes
    Often
    Very often

19. Do you feel attractive with your current back condition?

    Yes, very
    Yes, somewhat
    Neither attractive nor unattractive
    No, not very much
    No, not at all

20. Have you been a happy person during the past 6 months?

    None of the time
    A little of the time
    Some of the time
    Most of the time
    All of the time

(CONTINUED ON NEXT PAGE)

21. Are you satisfied with the results of your back management?

    Very satisfied
    Satisfied
    Neither satisfied nor unsatisfied
    Unsatisfied
    Very unsatisfied

22. Would you have the same management again if you had the same condition?

    Definitely yes
    Probably yes
    Not sure
    Probably not
    Definitely not

Thank you for completing this questionnaire. Please comment if you wish.

3-10-06

END

EQ-5D-Y (child, optional)

Describing your health TODAY

Under each heading, please tick the ONE box that best describes your health TODAY

Mobility *(walking about)*

I have no problems walking about ☐
I have some problems walking about ☐
I have a lot of problems walking about ☐

Looking after myself

I have no problems washing or dressing myself ☐
I have some problems washing or dressing myself ☐
I have a lot of problems washing or dressing myself ☐

Doing usual activities *(for example, going to school, hobbies, sports, playing, doing things with family or friends)*

I have no problems doing my usual activities ☐
I have some problems doing my usual activities ☐
I have a lot of problems doing my usual activities ☐

Having pain or discomfort

I have no pain or discomfort ☐
I have some pain or discomfort ☐
I have a lot of pain or discomfort ☐

Feeling worried, sad or unhappy

I am not worried, sad or unhappy ☐
I am a bit worried, sad or unhappy ☐
I am very worried, sad or unhappy ☐

How good is your health TODAY

- We would like to know how good or bad your health is TODAY.
- This line is numbered from 0 to 100.
- 100 means the best health you can imagine.
  0 means the worst health you can imagine.
- Please mark an X on the line that shows how good or bad your health is TODAY.

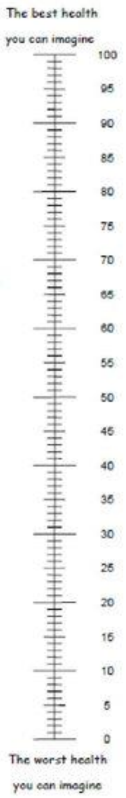

HOW-I-FEEL QUESTIONNAIRE

Developed by C.D. Spielberger, C.D. Edwards, J. Montuori, and R. Lushene

STAIC Form C-1

Name: ____ Age: _________ Date:_________

DIRECTIONS: A number of statements which boys and girls use to describe themselves are given below. Read each statement carefully and decide how you feel *right now*. Then put an X in the box in front of the word or phrase which best describes how you feel. There are no right or wrong answers. Don't spend too much time on any one statement. Remember, find the word or phrase which best describes how you feel right now, *at this very moment*.

1.	I feel	❑ very calm	❑ calm	❑ not calm
2.	I feel	❑ very upset	❑ upset	❑ not upset
3.	I feel	❑ very pleasant	❑ pleasant	❑ not pleasant
4.	I feel	❑ very nervous	❑ nervous	❑ not nervous
5.	I feel	❑ very jittery	❑ jittery	❑ not jittery
6.	I feel	❑ very rested	❑ rested	❑ not rested
7.	I feel	❑ very scared	❑ scared	❑ not scared
8.	I feel	❑ very relaxed	❑ relaxed	❑ not relaxed
9.	I feel	❑ very worried	❑ worried	❑ not worried
10.	I feel	❑ very satisfied	❑ satisfied	❑ not satisfied
11.	I feel	❑ very frightened	❑ frightened	❑ not frightened
12.	I feel	❑ very happy	❑ happy	❑ not happy
13.	I feel	❑ very sure	❑ sure	❑ not sure
14.	I feel	❑ very good	❑ good	❑ not good
15.	I feel	❑ very troubled	❑ troubled	❑ not troubled
16.	I feel	❑ very bothered	❑ bothered	❑ not bothered
17.	I feel	❑ very nice	❑ nice	❑ not nice
18.	I feel	❑ very terrified	❑ terrified	❑ not terrified
19.	I feel	❑ very mixed-up	❑ mixed-up	❑ not mixed-up
20.	I feel	❑ very cheerful	❑ cheerful	❑ not cheerful

Sample

HOW-I-FEEL QUESTIONNAIRE

STAIC Form C-2

Name: ____ Age: _________ Date:_________

DIRECTIONS: A number of statements which boys and girls use to describe themselves are given below. Read each statement carefully and decide if it is *hardly-ever*, or *sometimes*, or *often* true for you. Then for each statement, put an X in the box in front of the word that seems to describe you best. There are no right or wrong answers. Don't spend too much time on any one statement. Remember, choose the word which seems to describe how you usually feel.

1. I worry about making mistakes	❑ hardly-ever	❑ sometimes	❑ often
2. I feel like crying	❑ hardly-ever	❑ sometimes	❑ often
3. I feel unhappy	❑ hardly-ever	❑ sometimes	❑ often
4. I have trouble making up my mind	❑ hardly-ever	❑ sometimes	❑ often
5. It is difficult for me to face my problems	❑ hardly-ever	❑ sometimes	❑ often
6. I worry too much	❑ hardly-ever	❑ sometimes	❑ often
7. I get upset at home	❑ hardly-ever	❑ sometimes	❑ often
8. I am shy	❑ hardly-ever	❑ sometimes	❑ often
9. I feel troubled	❑ hardly-ever	❑ sometimes	❑ often
10. Unimportant thoughts run through my mind and bother me	❑ hardly-ever	❑ sometimes	❑ often
11. I worry about school	❑ hardly-ever	❑ sometimes	❑ often
12. I have trouble deciding what to do......................	❑ hardly-ever	❑ sometimes	❑ often
13. I notice my heart beats fast	❑ hardly-ever	❑ sometimes	❑ often
14. I am secretly afraid	❑ hardly-ever	❑ sometimes	❑ often
15. I worry about my parents	❑ hardly-ever	❑ sometimes	❑ often
16. My hands get sweaty	❑ hardly-ever	❑ sometimes	❑ often
17. I worry about things that may happen	❑ hardly-ever	❑ sometimes	❑ often
18. It is hard for me to fall asleep at night	❑ hardly-ever	❑ sometimes	❑ often
19. I get a funny feeling in my stomach.....................	❑ hardly-ever	❑ sometimes	❑ often
20. I worry about what others think of me	❑ hardly-ever	❑ sometimes	❑ often

sample

Physical Activity Questionnaire (Elementary School)

Name:_________________________ Age:___________

Sex: M_______ F_______ Grade:__________

Teacher:_______________________

We are trying to find out about your level of physical activity from ***the last 7 days*** (in the last week). This includes sports or dance that make you sweat or make your legs feel tired, or games that make you breathe hard, like tag, skipping, running, climbing, and others.

Remember:
1. There are no right and wrong answers — this is not a test.
2. Please answer all the questions as honestly and accurately as you can — this is very important.

---

1. Physical activity in your spare time: Have you done any of the following activities in the past 7 days (last week)? If yes, how many times? (Mark only one circle per row.)

	No	1-2	3-4	5-6	7 times or more
Skipping	❍	❍	❍	❍	❍
Rowing/canoeing	❍	❍	❍	❍	❍
In-line skating	❍	❍	❍	❍	❍
Tag	❍	❍	❍	❍	❍
Walking for exercise	❍	❍	❍	❍	❍
Bicycling	❍	❍	❍	❍	❍
Jogging or running	❍	❍	❍	❍	❍
Aerobics	❍	❍	❍	❍	❍
Swimming	❍	❍	❍	❍	❍
Baseball, softball	❍	❍	❍	❍	❍
Dance	❍	❍	❍	❍	❍
Football	❍	❍	❍	❍	❍
Badminton	❍	❍	❍	❍	❍
Skateboarding	❍	❍	❍	❍	❍
Soccer	❍	❍	❍	❍	❍
Street hockey	❍	❍	❍	❍	❍
Volleyball	❍	❍	❍	❍	❍
Floor hockey	❍	❍	❍	❍	❍
Basketball	❍	❍	❍	❍	❍
Ice skating	❍	❍	❍	❍	❍
Cross-country skiing	❍	❍	❍	❍	❍
Ice hockey/ringette	❍	❍	❍	❍	❍
Other:					
___________________________	❍	❍	❍	❍	❍
___________________________	❍	❍	❍	❍	❍

2. In the last 7 days, during your physical education (PE) classes, how often were you very active (playing hard, running, jumping, throwing)? (Check one only.)

- I don't do PE .... ❍
- Hardly ever .... ❍
- Sometimes .... ❍
- Quite often .... ❍
- Always .... ❍

3. In the last 7 days, what did you do most of the time *at recess*? (Check one only.)

- Sat down (talking, reading, doing schoolwork)...... ❍
- Stood around or walked around .... ❍
- Ran or played a little bit .... ❍
- Ran around and played quite a bit .... ❍
- Ran and played hard most of the time .... ❍

4. In the last 7 days, what did you normally do *at lunch* (besides eating lunch)? (Check one only.)

- Sat down (talking, reading, doing schoolwork)...... ❍
- Stood around or walked around .... ❍
- Ran or played a little bit .... ❍
- Ran around and played quite a bit .... ❍
- Ran and played hard most of the time .... ❍

5. In the last 7 days, on how many days *right after school*, did you do sports, dance, or play games in which you were very active? (Check one only.)

- None .... ❍
- 1 time last week .... ❍
- 2 or 3 times last week .... ❍
- 4 times last week .... ❍
- 5 times last week .... ❍

6. In the last 7 days, on how many *evenings* did you do sports, dance, or play games in which you were very active? (Check one only.)

- None .... ❍
- 1 time last week .... ❍
- 2 or 3 times last week .... ❍
- 4 or 5 last week .... ❍
- 6 or 7 times last week .... ❍

7. *On the last weekend*, how many times did you do sports, dance, or play games in which you were very active? (Check one only.)

None .... ❍
1 time .... ❍
2 — 3 times .... ❍
4 — 5 times .... ❍
6 or more times .... ❍

8. Which *one* of the following describes you best for the last 7 days? Read *all five* statements before deciding on the *one* answer that describes you.

A. All or most of my free time was spent doing things that involve little physical effort .... ❍

B. I sometimes (1 — 2 times last week) did physical things in my free time (e.g. played sports, went running, swimming, bike riding, did aerobics) .... ❍

C. I often (3 — 4 times last week) did physical things in my free time .... ❍

D. I quite often (5 — 6 times last week) did physical things in my free time .... ❍

E. I very often (7 or more times last week) did physical things in my free time .... ❍

9. Mark how often you did physical activity (like playing sports, games, doing dance, or any other physical activity) for each day last week.

	None	Little bit	Medium	Often	Very often
Monday	❍	❍	❍	❍	❍
Tuesday	❍	❍	❍	❍	❍
Wednesday	❍	❍	❍	❍	❍
Thursday	❍	❍	❍	❍	❍
Friday	❍	❍	❍	❍	❍
Saturday	❍	❍	❍	❍	❍
Sunday	❍	❍	❍	❍	❍

10. Were you sick last week, or did anything prevent you from doing your normal physical activities? (Check one.)

Yes .... ❍
No .... ❍

If Yes, what prevented you? ____________________________________

Insert ***External Letters of Permissions here*** *– such as letters from School Principals, Company CEOs, Charity Directors, Data Controllers for access to datasets/archives etc., Copyright permission for use of questionnaire if applicable*

[redacted]

Dear Garett and to whom it may concern,

I grant permission for McGowan Physio to be used as a recruitment site for Garett Van Oirschot's study into multimedia education for adolescent idiopathic scoliosis.

Please do not hesitate to contact me with any questions.

Yours sincerely,

[redacted]

[redacted]

Hi Garett,

Thank you for your contact. Yes, we are happy to help as best we can.
Of note, from past experience it can be a hard age to engage in research!
Forward on any relevant details and we can have a look.
Kind regards and best of luck,

[redacted]

The Scoliosis Advocacy Network

[redacted]

[redacted]

Hi Garett,

Thank you for your email. My apologies for the delay in getting back to you.
Yes, of course we would be glad to help with your research in any way we can.

Kind Regards,

[redacted]

Scoliosis Awareness & Support Ireland

Insert ***Local Research Ethics Approval Letters or Letters of Permissions to access databases****– such as letters from Hospitals, Nursing Homes, HSE Health Boards, Prisons, the HRCDC or any other body or organization that has a Research Ethics Committee)*

N/A

Insert ***Letter of endorsement from your supervisor*** *(if applicable) this should be a PDF which can either be included in this document or sent separately by email*

This was sent separately by email.

Insert a copy of ***Garda Vetting Certificate*** *(if applicable) this should be a PDF with which can either be included in this document or uploaded to Infohub separately, or sent by email.*

Garda Vetting request confirmed by UCD HR in below email for applicant (awaiting result for supervisor). Garda Certificate shown on next page.

Garda Vetting Application Process
Garda Vetting
To: Garett Van Oirschot

Inbox - UCD 13 March 2023 at 15:47

Dear Garett

This email is to confirm you are vetted from 11/03/2023 for 3 years.

Kind Regards
Rachel

Insert any ***other supporting documentation*** *that is not listed above here but is relevant to your study: For Example: a listing of support groups, a training programme for researchers, a debriefing doc, or a protocol for dealing with stressed participants or any data sharing/management agreements with other researchers (internal or external).*

Cover Letter Responding to Decision Points (*not required for a new submission – only use this section for your response to the committee review). When required, all responses should be emailed to* [redacted] *and must include the revised application form and the revised supporting documents above with revisions noted in red. Please cut and paste the decision points you received by email from the committee review and then answer each one in red ink which should correspond with the changes you made in red ink in either this document or the application form.*

Dear UCD Ethics Committee,

Thank you for your review points regarding this ethics application. Please find a response to each point below, as well as any revisions found in the body of the application or supporting documents in red.

Q5: Please define any abbreviations, like “MSK.” MSK = musculoskeletal; AIS = Adolescent Idiopathic Scoliosis; TAU = treatment as usual

Q5c) i: Please ensure and state explicitly that Qualtrics license will be in place for the duration of this research. Please state alternative platform should this not be the case. If Qualtrics license is due to expire then either consider using another platform from the start or explain how this may impact data collection and what contingencies are in place from transferring data collection across another platform. State data collection methods, i.e., experimental tasks, questionnaires, items etc. Describe the procedure clearly.
Since the last submission, the School of Public Health, Physiotherapy & Sports Science (SPHPSS) has altered plans and will now be operating research via Survey Monkey and the wording above has been changed to reflect this. The licence for this platform will be in use for the duration of the research project.
Data obtained from SurveyMonkey will include all baseline characteristics, responses to the multiple choice knowledge quiz, and the surveys for EQ-5D-Y, SRS-22r, STAI-Ch, PAQ-C.

Q5 ii: Please provide evidence of statistical power.
Information now included in Application Form.

Q5 v: These are not the methods of analysis but the data collection waves. Please state the methods of analysis. E.g. who will form the experimental and the control group, how randomization is going to be achieved, where/when the RCT protocol will be registered etc.
Information now clarified in Application Form

Q5e) ii: What is a no-stakes mcq - please clarify. This term was derived from previous work that used a multiple-choice exam to assess knowledge transfer (Doherty 2022, 10.1371/JOURNAL.PONE.0273007 and Doherty 2020 10.2196/19747). It means that the multiple choice exam score has no real-world implication for the participant.

Q5 ix: See comment on Q5c) i and report the total number of items for these psychometric tools. Are these psychometric tools appropriate for data collection from participants aged 10-18 years old? Have these been validated in these age cohorts? Please provide evidence either here or earlier in Q5c)i.
The EuroQOL-5Dimension-Youth (EQ-5D-Y) is the version that is recommended when assessing participants of ages 8-11 , but is also to be used for older cohorts that include adolescents in order to provide consistent results for a larger range that will include those who are younger and those who are adolescent (EuroQol Research Foundation. EQ-5D-Y User Guide, 2020. Available from: https://euroqol.org/publications/user-guides).

The Scoliosis Research Society 22-revised (SRS 22-r) is used for Adolescent Idiopathic Scoliosis and has been extensively used in the 10-18 age bracket and it is advised "All practitioners should utilize the SRS-22r, and its various translations, as it is the most recent and most validated version of the SRS Patient Based Outcomes Questionnaire" as noted on: https://www.srs.org/professionals/online-education-and-resources/patient-outcome-questionnaires

Q6: An ethical issue that has not been addressed is that some children and adolescents can be upset and distressed by questioning around mental health states and quality of life.
Questioning around mental health status does occur in the STAI-CH or certain domains of the EQ-5D-Y and the SRS-22 and should a participant report feeling upset or distressed, then the author will be available to answer any questions or concerns that have arisen. Should further support be required, then a referral can be made to the participant's GP, orthopaedic consultant, physiotherapist, as well as a support network such as www.spunout.ie, www.jigsaw.ie, or a direct referral to a psychologist known for working with child and adolescent scoliosis (https://www.app.therapyhub.ie/therapist/deirdre-walsh).

Q7a): A 10-year old child is not an adolescent. Adolescents are typically considered the age period of 12-17 years old. This is an important issue in the application and either the target group should be revised or it should be argued why there is a need to include children as young as 10 years old. Further, if some participants are 18 years old then a different recruitment and consent procedure should be followed as these are no longer considered minors and they can provide consent themselves.
It is acknowledged that 10-year-olds are not considered adolescents. The wording has been changed in the protocol to include 'child and adolescents' where applicable. The research literature surrounding 'Adolescent Idiopathic Scoliosis' repeatedly and consistently includes participants in the 10-18 age bracket, due to the nature of the condition where early signs of AIS are present in 10-year-olds and treatment lasting into late adolescence. This has created a field of healthcare research dedicated to the 10-18 year old AIS cohort, and while the nomenclature is not technically accurate compared to 'child' or 'adolescent' classification in other healthcare domains, it is the common practice in this area.
The applicant is attempting to remain consistent with current practice by including this age range in this study, but is happy to ensure 'child & adolescent' is used instead of just 'adolescent' in order to avoid such confusion. The recruitment procedures will be updated to ensure that 18 year olds are provided with the appropriate consent forms.

Q7b) i: "Awaiting response from two scoliosis consultant offices as well as the Scoliosis Awareness & Support Ireland". Please supply these in the supporting documents when available. Scoliosis Awareness & Support Ireland has sent approval since this application was last submitted. See copy of email above.

Q7b): does recruitment from private physiotherapy practice require audit and review of client profiles? Are these all current patients of the private practice and, if so, how is a continuing relationship between the patient and the practice defined? Does the contract governing the physiotherapist-patient relationship allow the physiotherapist to approach the patient directly to tell them about the research?
No, recruitment does not require the applicant to audit patient profiles. The practice did have its own internal statistic of the number of scoliosis patients in the last 12 months but this number was determined by the clinic without any UCD involvement. The recruitment from this physiotherapy practice would involve current or future patients becoming aware of the study via posters, websites, social media, and word of mouth from staff or other patients.
Recruitment would occur from the current and future patients of the practice, until study recruitment is terminated, but the patients would be referred to the online recruitment process and the continuing relationship between the clinic and patient would proceed as normal, with the relationship defined by the need for progression and supervision of exercises, instructions from the orthopaedic consultant, discretion of the patient/family, and discretion of the treating therapist and this relationship would continue irrespective of participation in this study.
The practice regularly advertises research in Ireland that pertains to the scoliosis population and the governing guidelines from CORU, the state regulator, permit physiotherapists to pass along such information. Such CORU guidelines for patient protection would also pertain to the applicant, a practicing physiotherapist.

Q7b) ii: Are adolescents and children younger than 18 years going to be approached by the applicant? The procedure should be through their parents/guardians who will first need to access the information sheet and if they consent, then should pass on any materials to children, who in turn have to assent before taking part.

No, approaches will only be made to parents or those 18years of age.

Q8g): does the researcher currently treat some of the participants (i.e. those recruited from the private physiotherapy practice)? Consider the ethical aspects of knowing some participants in clinical detail but not knowing others. What are the implications of this for research design?
There is potential that the applicant could treat a small number of participants, but in such an event would still be blinded to the intervention group assignment because the interventional videos are being sent out by the supervising author instead of the applicant.
There are no in-person outcome measures that require in-person attendance in this study, so the study does not mandate the applicant to interact with any of the participants beyond the recruitment process and answering any questions/concerns. Thus the study would run in parallel to any clinical attendance.

Q9: The potential of distressing or upsetting some children, adolescents and young adults has not been considered. How do researchers ensure that their young participant who may become distressed by

participating in their study will be provided adequate support? How will they be debriefed? Please provide all this information along with a distress protocol.
As per Q6, applicant will make himself available to address concerns and supply the resources mentioned above in Q6 if required.

Q10d) & e): Covid is a risk of face-to-face interactions that should be acknowledged and controlled, and noted in the PIL risks.
If recruitment occurs via in-person / word of mouth, it will be in a healthcare facility already subject to all current HSE Covid protection guidelines. Otherwise the online nature of recruitment and nature of the study should mitigate this risk.

Q16: Please state how the data will be anonymised – e.g. will a code be assigned to each subject, and the link to their name not retained?
Now clarified in PIL

Supports:

Please describe the content of the videos in the information sheets for both the participants and parents/guardians. If all information content comes from Scoliosis Research Society I'm sure the participants and guardians will feel assured to know that. Describe in what way the UCD-made videos will differ from the standard ones.
The UCD-made videos will present two versions of the Scoliosis Research Society videos. In one intervention the videos will simply follow the same script and graphics as the Scoliosis Research Society. In the second intervention, the videos will still use the same script but will use graphics in accordance with the Cognitive Theory of Multimedia Learning and other recommendations on multimedia education from the literature (for example, using images alongside narrative descriptions, using seductive music, avoiding duplication of words + images, etc).

Info leaflet for patients: Please give more detail in "What happens if I volunteer?" how many of video content must they watch per week and for how many weeks? "answer some questions every few months" - questions about what, and how many months?
This is now clarified about the 5-item quiz every week for six weeks, as well as the 30 questions follow-up at the end of the six weeks and again at six months

Information Sheet: should inform that the right to withdraw will cease once the data is irrevocably anonymized, and the consents should ask permission to do this.
Form amended to reflect this.

Please provide us with drafts not only of the physical materials to advertise the study but also drafts of the digital materials for social media.
Inserted above

Parent/guardian information sheet:
This is addressed to "you", but it should be addressed to the parent/guardian.
Terminology now corrected.

Section on how privacy will be protected is not very clear. Is a parent likely to be knowledgeable about GDPR regulations and their implications for research? Clarify what is meant by keeping data confidential and "made anonymous to the researcher". It is not correct to say that the data cannot be traced back to the participant as anyone with access to the code/ID numbers can reconcile the data with identity of participant. Please specify the practical steps that are to be taken to maintain privacy and confidentiality.
Clarified above in Q.14

Section on conflict of interest identifies a conflict of interest (physiotherapist treats participants) but does not explain why this is a conflict or what steps (if any) are taken to remedy the conflict.

Page 1: incomplete, please check the appropriate boxes.
Complete

Participant information sheet: If there will be participants aged 18 or older they will need a separate one than the one for minors.
Corrected and shown above.

In both sheets the risk of getting upset should be disclosed to participants. Include appropriate sources of mental health support or other supports appropriate for children and young adults e.g., spunout.ie in the information sheet.
Clarified in all information sheets and listed above in red.

In the minor's sheet it should be very clear that they don't have to take part even if their parents agreed to do so.
Have added this to the participant assent form.

Please provide adequate information about this study to young people who wish to participate e.g., there is no mention that they will fill in questionnaires or how long it will take.
Have added this information to both information sheets above in red.

A randomised controlled trial is the research design, implies there is an intervention and control group. What is the difference in what education the two groups receive? The control group will continue to receive treatment as usual, which includes the education that is received from their usual healthcare providers. The two treatment groups will receive educational videos containing identical information, but differing in design characteristics (with modifications to background music, graphics, segmentation of information, speaking rate, to name a few).

Is this a purposeful sample? Where and how does randomisation occur? There is no reference to randomisation in PIL/consent forms
The different treatment arms are now better clarified in the information forms.

Will all participants receive equal material at the end?
All participants will receive equal educational material in the end: approximately 3 minute videos with the same script and information recited.

If the data is collected via Qualtrics it is anonymous and therefore cannot be withdrawn when submitted
As noted above, the data will now be collected via Survey Monkey due to changes within the School of PHPSS. Yes, the anonymity and inability to withdraw is now clearly noted in the consent forms.

The parent PIL should be rephrased to relate to their child participating and not themselves.
Completed and marked in red above.

Please ensure all permissions are granted before proceeding.
